# An Approach to Accelerate Healing and Shorten the Hospital Stay of Patients With Anastomotic Leakage After Esophagectomy: An Explorative Study of Systematic Endoscopic Intervention

**DOI:** 10.3389/fonc.2021.657955

**Published:** 2021-05-17

**Authors:** LeQi Zhong, JiuDi Zhong, ZiHui Tan, YiTong Wei, XiaoDong Su, ZheSheng Wen, TieHua Rong, Yi Hu, KongJia Luo

**Affiliations:** ^1^ Sun Yat-Sen University Cancer Center, State Key Laboratory of Oncology in South China, Collaborative Innovation Center for Cancer Medicine, Guangzhou, China; ^2^ Department of Thoracic Suegry, The First Affiliated Hospital of Guangxi University of Chinese Medicine, Nanning, China; ^3^ Guangdong Esophageal Cancer Institute (GECI), Guangzhou, China

**Keywords:** esophageal cancer, anastomotic leak, endoscopic intervention, clips, sealants, perioperative complications

## Abstract

**Objective:**

To explore the comprehensive role of systemic endoscopic intervention in healing esophageal anastomotic leak.

**Methods:**

In total, 3919 consecutive patients with esophageal cancer who underwent esophagectomy and immediate esophageal reconstruction were screened. In total, 203 patients (5.10%) diagnosed with anastomotic leakage were included. The participants were divided into three groups according to differences in diagnosis and treatment procedures. Ninety-four patients received conventional management, 87 patients received endoscopic diagnosis only, and the remaining 22 patients received systematic endoscopic intervention. The primary endpoint was overall healing of the leak after oncologic esophageal surgery. The secondary endpoints were the time from surgery to recovery and the occurrence of adverse events.

**Results:**

173 (85.2%; 95% CI, 80.3-90.1%) of the 203 patients were successfully healed, with a mean healing time of 66.04 ± 3.59 days (median: 51 days; range: 13-368 days), and the overall healing rates differed significantly among the three groups according to the stratified log-rank test (P<0.001). The median healing time of leakage was 37 days (95% CI: 33.32-40.68 days) in the endoscopic intervention group, 51 days (95% CI: 44.86-57.14 days) in the endoscopic diagnostic group, and 67 days (95% CI: 56.27-77.73 days) in the conventional group. The overall survival rate was 78.7% (95% CI: 70.3 to 87.2%) in the conventional management group, 89.7% (95% CI: 83.1 to 96.2%) in the endoscopic diagnostic group and 95.5% (95% CI: 86.0 to 100%) in the systematic endoscopic intervention group. Landmark analysis indicated that the speed of wound healing in the endoscopic intervention group was 2-4 times faster at any period than that in the conservative group. There were 20 (21.28%) deaths among the 94 patients in the conventional group, 9 (10.34%) deaths among the 87 patients in the endoscopic diagnostic group and 1 (4.55%) death among the 22 patients in the endoscopic intervention group; this difference was statistically significant (Fisher exact test, P < 0.05).

**Conclusion:**

Tailored endoscopic treatment for postoperative esophageal anastomotic leakage based on endoscopic diagnosis is feasible and effective. Systematic endoscopic intervention shortened the treatment period and reduced mortality and should therefore be considered in the management of this disease.

## Introduction

As the seventh most commonly diagnosed cancer, esophageal carcinoma (EC) is associated with a dismal fatality rate, ranking as the sixth most common cause of cancer-related death (1). Once esophageal cancer is confirmed, radical resection is typically recommended, as it is of the most effective therapeutic approaches for select patients. Despite the considerable improvement in surgical conditions and skills, however, esophagectomies are still associated with various complications, of which anastomotic leakage is a disastrous postoperative complication that seriously affects patient quality of life due to both its high incidence (5-40%) and associated mortality (2-60%) (2–9). As a consequence, improvements in leak management are of vital necessity to reduce overall mortality.

Leaks after esophagectomy are defined as full-thickness gastrointestinal defects involving the esophagus, anastomosis, staple line, or conduit irrespective of the presentation or method of identification. Along with the development of esophageal anastomotic leakage (EAL), one consequence followed close on the heels of another. Firstly, EAL is the greatest risk factor for perioperative complication-related death, with up to 60% mortality rates, and the risk of death for patients with EAL is 3 times higher than that for those without EAL (7–11). Moreover, in the short run, it increases the length of hospital stay, prolongs the oral feeding time, contributes to the risk of anastomotic hemorrhage, and increases risk of reoperation. In the long run, a positive association between the occurrence of anastomotic stricture and the development of EAL was observed. EAL can also impair long-term survival, negatively impact surgical and oncologic outcomes and be related to cancer recurrence after surgical resection for esophageal malignancy (5, 7, 9–11).

The diagnosis or interference time of EAL explains the severity of this complication; more specifically, the most predominant risk factors for the subsequent clinical outcome are the patients’ delay as well as the delay of diagnosis or even the absence of any interference, so it is undisputed that prompt diagnosis and immediate intervention are of vital significance to prevent further damage and to control the ensuing clinical development. Throughout the course of intervention, the use of a multidisciplinary diagnostic and treatment approach is undoubtedly highly important.

Traditionally, there are several methods used to detect EAL, of which routine contrast medium esophagography is widely utilized and has gained international recognition. In addition, direct surgical exploration, oral administration of methylene blue, and CT scans with or without oral contrast are extensively used. However, there is no consensus within the literature with regard to whether, when or which strategy should be used, even though their limitations are well documented. Some researchers have suggested that the routine use of contrast radiography be suspended, since it can be unreliable in the detection of anastomotic leaks, with a reported sensitivity between 40% and 66%, and aspiration pneumonia due to aspiration of the contrast agent was noted (12, 13). Meanwhile, operative exploration is limited by its high mortality rate; oral methylene blue may not be proper for diagnosing late EAL, as adhesions formed after esophagectomy may result in localized collection of the dye, making it difficult to identify EAL; and computed tomography (CT) scanning does not provide information about gastric conduit viability, so early ischemic or necrotic areas could be missed (12–16). As endoscopic techniques have begun to be applied clinically over the past decade, they have shown clear advantages (e.g., direct visualization and quantification of the defect, ability to determine gastric conduit viability, and both the sensitivity and specificity could reach up to 95-100%) (14–16).

In terms of treatment, the therapeutic strategies for this issue range from palliative treatment such as antibiotics and nutritional support to operative exploration and endoscopic management using stents, clips, glues, etc., or their combination. All of these efforts share the same goal: to close the breach and eliminate contamination. Traditional surgical repair has certain disadvantages, such as increased hospitalization costs and mortality and extended hospital stays, which obviously conflict with the notion of rapid rehabilitation surgery. Fortunately, minimally invasive endoscopic therapies may have advantages such as enhanced safety, minimal invasiveness, quicker recovery, lower treatment cost, etc., when compared with traditional open surgical methods (14–16).

However, clinicians remain reluctant to perform endoscopy after esophagectomy because of the theoretical risk of disrupting the anastomosis or worsening the EAL (17–19). In our cases, endoscopic intervention was found to aid in making a precise diagnosis and in deciding the most appropriate clinical strategy without increasing the incidence of complications and mortality. As highlighted in a recently published Position Statement of the European Society of Gastrointestinal Endoscopy, it is important to have a systematic approach for the diagnosis and treatment of GI perforations (20). Therefore, this investigation proceeded with the aim of evaluating the safety and efficacy of this new approach to diagnosing and treating anastomotic fistula and to assess the role of endoscopic intervention throughout the entire rehabilitation process of EAL.

## Patients and Methods

**Figure 1:**
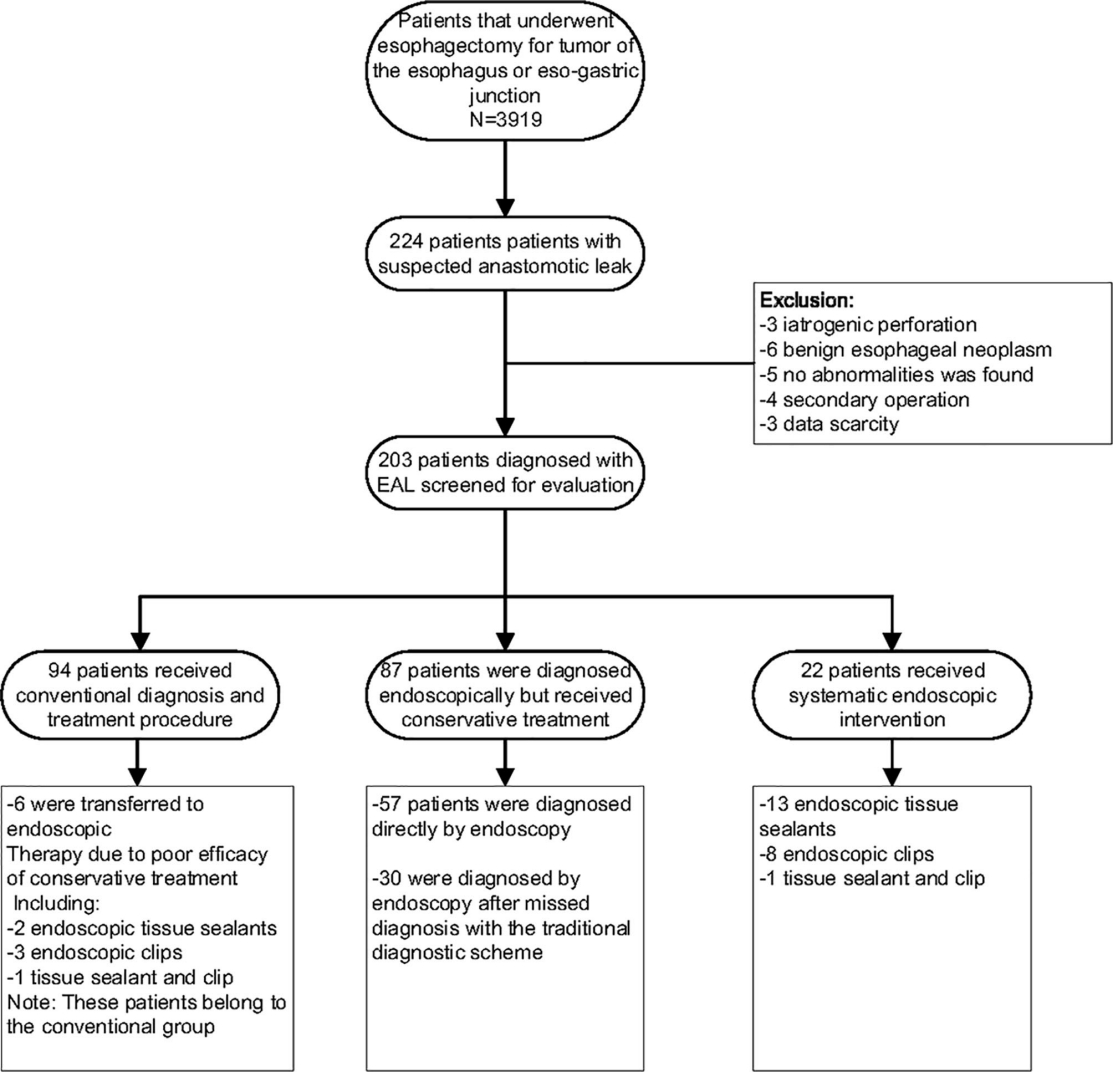
Description of the selection of the studied population of patients.

This was a single-center retrospective study conducted at our Thoracic Surgery Department. We analyzed our clinical databank and screened out all suspected EAL patients who had undergone esophagectomy between January 2012 and August 2019 at the Sun Yat-Sen University Cancer Center. To improve the homogeneity between the study groups, only patients with anastomotic leakage after esophagectomy due to malignant esophageal tumors were included. Other esophageal leaks, such as iatrogenic leakage, EAL from benign esophageal disease or following gastrectomy, were excluded. Other exclusion criteria were a prior history of esophageal surgery, cases managed by primary surgery, operation performed at another institution and incomplete medical records. The specific process of patient enrollment is shown in the flow chart (**Figure 1**).

Records were reviewed to collect patient demographics, tumor characteristics, preoperative chemoradiotherapy information, surgical procedures, diagnostic methods, leakage therapy regimens, clinical outcomes, mortality and complications.

### Surgical Characteristics

A total of 3919 patients underwent esophagectomy during the evaluation period, of whom 203 were confirmed to have EAL and were included in the analysis (**Figure 1**). Among this population, 138 patients underwent open surgery, including 57 patients who underwent surgery according to the Sweet procedure, 61 patients who underwent surgery according to the McKeown procedure, and 20 patients who underwent Ivor-Lewis surgery. The remaining 65 patients underwent minimally invasive esophagectomy procedures such as the mediastinoscopic transmediastinal approach (n=3), thoraco-laparoscopic McKeown (n=42) and Ivor-Lewis (n=3) esophagectomy, and robot-assisted McKeown (n=17) with the aid of the da Vinci^®^ system (Intuitive Surgical, Sunnyvale, CA). Construction was completed in 201 patients by gastric conduit and in 2 patients by colon interposition. The decision of surgical modality was made at the discretion of the surgeon performing the operation according to the patient’s actual condition.

### EAL Diagnosis and Intervention

Radiological contrast studies or endoscopy were routinely performed to screen for the existence of possible leakage at approximately day 7 after surgery. Once EAL was confirmed, the surgeon responsible for the respective case would decide on a treatment plan and initiate intervention. The specific diagnoses and intervention procedures of the 203 included patients are presented in **Figure 1**.

### Conservative Treatment

Conservative approaches included nutritional support, gastrointestinal decompression through an intraoperatively placed gastric tube, perianastomotic drainage *via* a surgically placed prophylactic chest tube and systemic antibiotics. Supplemental nutritional support was generally provided through a preplaced jejunal nutrition tube during esophagectomy and occasionally through total parenteral nutrition support. Proton pump inhibitor (PPIS)-aided therapy was also included for gastrointestinal decompression, and the intraoperative indwelling gastric tubes were not pulled out until the anastomotic leakage healed. The leak cavity was flushed several times with irrigation fluids containing gentamycin in saline, with the same purpose as thoracic drainage to clear most of the pus. In accordance with the irrigation regimen, all patients received intravenous broad-spectrum antibiotics (2, 5, 9, 11).

### Endoscopic Intervention

Endoscopic interventions were performed by veteran endoscopic surgeons. The endoscopic strategy was subdivided into two types: diagnostic and therapeutic. The location of the anastomosis and the lesion, the extent of the orifice, and the presence of pus were confirmed and evaluated during the diagnostic phase. Then, the leaks were subdivided into the following categories according to the Esophagectomy Complications Consensus Group (ECCG) classification (21):

Type I: Local defect requiring no change in therapy or treated medically.

Type II: Localized defect requiring interventional but not surgical therapy.

Type III: Localized defect requiring surgical therapy.

Then, the therapeutic phase was carried out at the discretion of the responsible surgeon according to the EAL characteristics found above. First of all, in the course of endoscopic intervention, whether the EAL was infected or not would be one of our major focuses. If the EAL was infected, we would irrigate the pus cavity with normal saline under the navigation of ultrafine gastroscopy, and then immediate suction and irrigation of the abscess cavity would be established by Endoscopic Trans-nasal Inner Drainage. Generally, the pus would exterminate in about 7-14 days, subsequent systematic endoscopic therapies would be carried out based on the status of patients and the results of endoscopic reexamination, the processes of drainage was not counted in the number of endoscopic sessions. If the EAL was not infected, the systematic endoscopic treatment would administrate directly. Treatment strategies included a ‘wait and see’ strategy (endoscopic diagnostic group), administration of tissue sealant, the use of an endoscopic clip or the application of combined therapy (endoscopic intervention group). Endoscopic treatment was systematically performed until an effective outcome was achieved or the patient died (6).

Details of intervention strategies in systematic endoscopic group is described as follows.

During the diagnostic procedure or the reexamination process, the depth of lesion was confirmed. If the depth of the wound was less than 1cm, the patient would be treated by endoscopic clips; otherwise, by biological tissue sealants to avoid the formation of residual cavity.

#### Endoscopic Clips

The first clip is proposed to place through the most distal part of the leak to the oral side successively as this prevents accidental snagging and drooping. Then, flushing the anastomosis with normal saline and observing whether there are bubbles, so as to judge whether it is closed completely. Lastly, checking the tension of the anastomosis. If the tension is high or the closure is incomplete, endoscopic review and following sessions will be administrated 7 days later.

#### Endoscopic Tissue Sealants

Firstly, we use a small endoscopic brush to clean the wound and make it bleed slightly, then spray sealants to fill the fistula and to stop the bleeding. The biological sealants consist two components, one component consists of the antifibrinolytic solution (aprotinin) and a protein concentrate (fibrinogen) derived from human plasma, and the other component includes human thrombin (or a bovine thrombin) and a calcium chloride solution. The two solutions are delivered in a dual-barrel syringe and combined at the site of desired application, through a double lumen catheter, to form a firm, white, rubber-like mass with strong adhesive properties within few seconds of being mixed.

### Endpoints

The primary endpoint was the overall healing of leakages after oncologic esophageal surgery. Complete healing of the EAL was defined as patient recovery (no abnormality after oral feeding) after assessment with endoscopy or *via* follow-up X-ray or CT contrast study. The secondary endpoint was the time (in days) from surgery to recovery and the occurrence of adverse events (sinus formation, bleeding, anastomotic stenosis, etc.). Failure was defined as death or loss to follow-up.

### Statistical Analysis

Primary data were managed and extracted from the hospital data management system and then analyzed by IBM SPSS Statistics version 25.0 (Inc, Chicago, Illinois, USA). Continuous variables are presented as the means ± standard deviations (SD), and categorical data are presented as numbers and percentages. Multivariable analyses with the Cox proportional hazards model were used to estimate the simultaneous effects of prognostic factors on healing. All eligible patients were included in the analysis of overall healing by the Kaplan–Meier method and the log-rank test to calculate corresponding *P* values. First, a univariate analysis using various factors associated with EAL healing time was performed. Next, to identify significant independent factors related to the time needed for EAL healing, multivariate Cox regression analysis was performed using factors identified as significant variables and selected potential confounding factors from the univariate analysis. Given that all patients in the endoscopic intervention group had healed within 90 days after surgery (except for one death on day 90), to explore the role of endoscopic technology in the healing process of EAL at different time periods from esophagectomy to rehabilitation, an exploratory analysis based on the landmark analysis method was performed according to landmark points of 30 days, 60 days, 90 days, and post-90 days, with the hazard ratio calculated separately for events that occurred each month after grouping and events that occurred between 90 days and the end of the follow-up period (22–24). We then performed a test for the interaction between treatment and time. In all time-to-event analyses (i.e., overall and landmark), for each type of event, data were censored at the time of the first event that occurred in a patient. Additionally, all patients were included in the complication assessment. Differences were considered to be statistically significant when the *P* value was 0.05 or less. All statistical tests were two-sided.

## Results

### Patient Characteristics

A total of 224 patients were suspected of having EAL due to esophagectomy during the study period, of whom 21 were excluded from the study (**Figure 1**). The remaining 203 patients were included in the analysis (**Table 1** shows the baseline clinical data). Among these 203 patients, 94 patients received conventional diagnosis and treatment procedures (conventional group); of the other 109 patients, 87 patients (including one patient for whom endoscopic clipping was attempted but failed) were diagnosed endoscopically but received conservative treatment (endoscopic diagnostic group), and 22 patients were diagnosed and treated by endoscopy directly (endoscopic intervention group) (**Figure 1**). There was no significant difference in clinical baseline among the three groups except age (**Table 2** shows the comparison of clinical characteristics according to the diagnosis and treatment procedures).

**Table 1:** Basic characteristics of the 203 patients with EAL and Determinants of EAL postoperative overall healing in patients with EAL.

Factors	No. of patients	Univariate	Multivariate		
	(N=203)	*P* value	HR	95% CI	*P* value
**Diagnosis and treatment procedure**		**<0.001**			
conventional management^a^	94				
endoscopic diagnosis	87		1.67	1.20-2.32	0.002
systematic endoscopic intervention	22		2.81	1.70-4.63	<0.001
**Sex** (male vs. female)	167/36	0.734			
**Age** (year) (<62 vs. ≥62)	102/101	0.818			
**Body mass index** (kg/m2) (<22 vs. ≥22)	108/95	0.846			
**ASA PS** (II vs. III)	151/52	0.663			
**Smoking index**(package*years)(<25 vs. ≥25)	101/102	0.325			
**Drinking history** (yes vs. no)	69/134	0.949			
**Diabetes Mellitus** (yes vs. no)	23/180	0.120			
**Hypertension** (yes vs. no)	52/151	0.171			
**Gastric ulcer and/or gastritis** (yes vs. no)	144/59	0.485			
**Tumor location** (upper vs. middle vs. lower thoracic)	21/124/58	0.074			
**Tumor staging** (I vs. II vs. III vs. IV)	44/62/87/10	0.113			
**Minimally invasive surgery** (yes vs. no^a^)	65/138	**0.003**	1.55	1.11-2.15	0.009
**Postoperative fever** (yes vs. no)	60/143	0.112			
**Neo-adjuvant therapy** (yes vs. no)	30/173	0.846			
**Leak location** (cervical vs. intrathoracic)	123/80	0.686			

HR, Hazard ratio; 95% CI, 95% confidence interval; ASA PS, American Society of Anesthesiologists classification of physical status.

a Reference category. Bold values means the difference was statistically significant.

**Table 2:** Comparison of clinical characteristics according to the diagnosis and treatment procedures.

Variables	Conventional management	Endoscopic diagnosis	Systematic endoscopic intervention	P value
**Male**	71(75.5%)	78(89.7%)	18(81.8%)	0.045
**Age (years)**	59.5	63.2	61.8	0.005
**Body mass index (kg/m2)**	22.3	21.5	22.0	0.211
**Smoking index**	507.6	497.2	429.6	0.802
**Drinking history**	32(34.0%)	30(34.5%)	7(31.8%)	0.972
**Diabetes Mellitus**	7(7.4%)	12(13.8%)	4(18.2%)	0.218
**Hypertension**	25(26.6%)	23(26.4%)	4(18.2%)	0.699
**Gastric ulcer and/or gastritis**	65(69.1%)	62(71.3%)	17(77.3%)	0.749
**Tumor location**				0.545
**upper thoracic**	12(12.8%)	6(6.9%)	3(13.6%)	
**middle thoracic**	58(61.7%)	52(59.8%)	14(63.6%)	
**lower thoracic**	24(25.5%)	29(33.3%)	5(22.7%)	
**Tumor staging**				0.058
**I**	13(13.8%)	23(26.4%)	8(36.4%)	
**II**	31(33.0%)	29(33.3%)	2(9.1%)	
**III**	46(49.0%)	30(34.5%)	11(50.0%)	
**IV**	4(4.2%)	5(5.7%)	1(4.5%)	
**Postoperative fever**	27(28.7%)	28(32.2%)	5(22.7%)	0.666
**Neo-adjuvant therapy**	11	16	3(13.6%)	0.443
**Anastomosis infection**	Unavailable	52(59.8%)	15(68.2%)	0.469
**Leak size(mm^2^)**	Unavailable	52.34	52.09	0.992

### Diagnosis

Traditional radiological contrast studies (n=124) resulted in 30 missed diagnoses (omission diagnostic rate=24.19%) and 5 misdiagnoses among the EAL patients; hence, the sensitivity of traditional diagnostic methods was 75.81%. Comparatively, endoscopy correctly diagnosed the remaining 79 patients who underwent endoscopic examination directly due to suspected EAL with 100% accuracy. Moreover, endoscopy not only correctly identified the 5 false-positive patients from the radiological contrast study but also detected the 30 leaks that were missed.

### Overall Healing

EAL was treated during hospitalization for all patients, and 173 (85.2%; 95% CI: 80.3-90.1%) of them successfully healed, with a mean healing time of 66.04 ± 3.59 days (median: 51 days; range: 13−368 days). The overall healing rates in the three groups differed significantly based on the results of the stratified log-rank test (*P*<0.001).

**Table 3** shows the characteristics of EAL of the 22 study patients who underwent systematic endoscopic intervention.

**Table 3:** Characteristics of EAL of the 22 study patients who underwent systematic endoscopic intervention.

Patient	Age and sex	Time to diagnosis (Days)		Location of anastomosis	Opening size (mm)	Infection of anastomosis	Number of sessions	Heling time (Days after surgery)	Clinical Outcome		Complication
**13 Endoscopic sealants**											
1	63; Male	7		Intrathoracic	10*9	yes	1(refusing following sessions)	90	Died		Died
2	57; Male	8		Intrathoracic	10*10	yes	1(refusing following sessions)	85	Discharged		None
3	69; Male	7		Intrathoracic	15*10	yes	3	73	Discharged		None
4	60; Male	7		Cervical	8*6	yes	2	52	Discharged		None
5	62; Male	7		Cervical	8*8	yes	2	51	Discharged		None
6	54; Male	7		Intrathoracic	3*3	no	4	44	Discharged		None
7	44; Male	10		Intrathoracic	4*4	no	3	37	Discharged		None
8	59; Male	7		Cervical	12*10	yes	1	36	Discharged		Stenosis
9	73; Male	4		Cervical	2*2	no	2	28	Discharged		None
10	57; Male	9		Intrathoracic	7*5	no	2	23	Discharged		None
11	50; Male	8		Intrathoracic	5*5	no	2	22	Discharged		None
12	66; Female	10		Intrathoracic	5*5	no	2	15	Discharged		None
13	50; Male	8		Intrathoracic	2*2	no	1	15	Discharged		None
**8 Endoscopic Clips**											
14	61; Female		7	Cervical	7*5	yes	2	78	Discharged	None	
15	69; Male		7	Cervical	10*10	yes	3	69	Discharged	None	
16	54; Male		7	Cervical	8*8	yes	2	46	Discharged	None	
17	72; Male		8	Intrathoracic	15*3	no	4	38	Discharged	None	
18	75; Male	7		Intrathoracic	4*4	yes	1	36	Discharged		None
19	71; Female	7		Intrathoracic	3*3	yes	2	35	Discharged		None
20	66; Female	7		Cervical	7*5	yes	1	33	Discharged		None
21	63; Male	7		Cervical	10*8	yes	1	32	Discharged		None
**1 Combination Therapy**											
22	65; Male	7		Intrathoracic	12*6	yes	1	39	Discharged		None

Length *(and) Width.

The median healing time of EAL was 37 days (95% CI: 33.32-40.68 days) in the endoscopic intervention group, 51 days (95% CI: 44.86-57.14 days) in the endoscopic diagnostic group, and 67 days (95% CI: 56.27-77.73 days) in the conventional group (**Table 4**).

**Table 4:** Overall healing the 203 patients with EAL.

	No. of patients	Healing rate	Median Healing time (95%CI)
Groups	(N=203)	*P*<0.001	
Conventional management	74/94	82.2%	67(56.27-77.73 Days)
Endoscopic diagnosis	78/87	89.7%	51(44.86-57.14 Days)
Systematic endoscopic intervention	21/22	95.5%	37(33.32-40.68 Days)
Total	173/203	85.2%	54(49.79-58.21 Days)

CI, Confidence Intervals.

**Figure 2:**
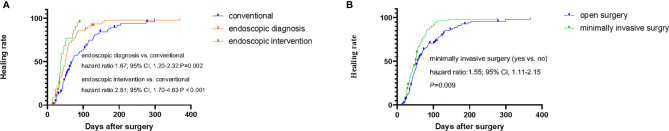
**(A, B)** Kaplan–Meier curves for independent predictors of EAL healing.

The univariate analysis showed a significant relationship between diagnosis and treatment procedure (conventional management vs. endoscopic diagnosis vs. systematic endoscopic intervention) and minimally invasive surgery (yes vs. no) (**Table 1**). Cumulative healing rates after surgery calculated with the Kaplan−Meier method and stratified by these significant factors are shown in **Figures 2A, B**.

The multivariate analysis results demonstrated that diagnostic and treatment procedures (conventional management vs. endoscopic diagnosis vs. systematic endoscopic intervention) and minimally invasive surgery (yes vs. no) were significant independent factors for EAL healing time (P<0.001 and P=0.009, respectively) (**Table 1**).

### Landmark Analysis

**Figure 3:**
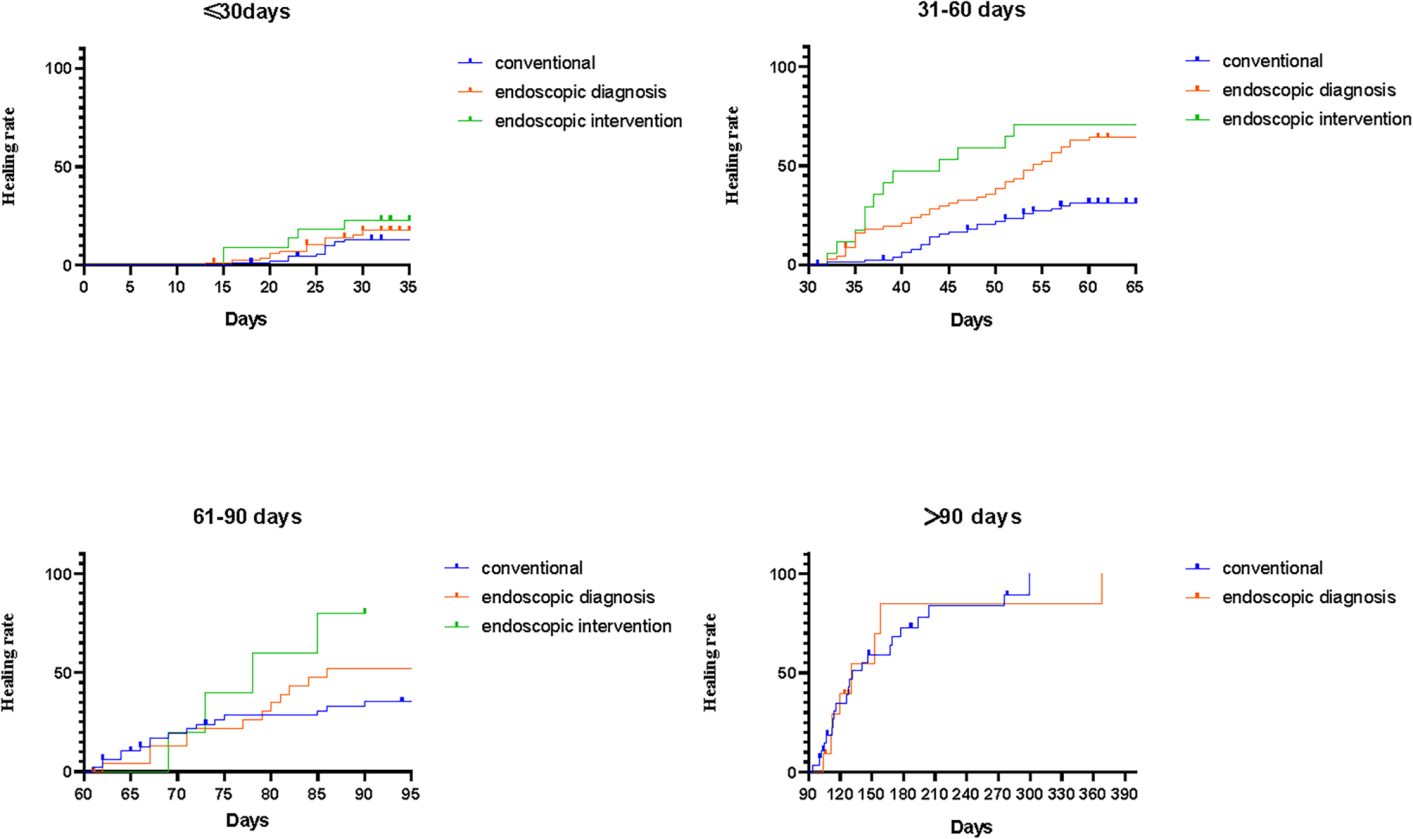
The survival curves of Interventional vs. Endoscopic groups at different landmark period.

**Figure 4:**
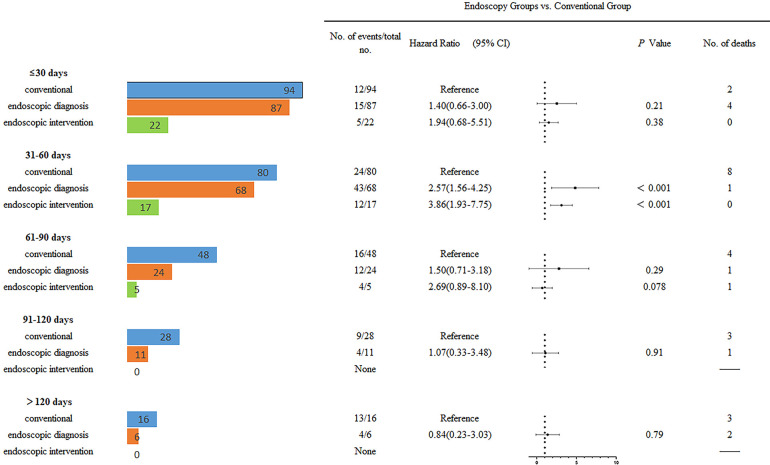
Overall healing of patients with EAL based on landmark analysis and corresponding hazard ratios. The number of unhealed patients with EAL and the corresponding hazard ratios are shown at various time points for the groups. A total of 94 patients in the conventional group, 87 in the endoscopic diagnosis group, and 22 in the endoscopic intervention group; the corresponding numbers at 60 days were 48, 24, and 5, and the corresponding numbers at 90 days were 28, 11, and 0.

The landmark analysis results indicated that the speed of wound healing in the endoscopic intervention group was faster than that in the conservative group at any period. The healing characteristics of the different groups at various landmark periods are illustrated in **Figures 3** and **4**. It was not difficult to find that the healing speed of the endoscopic intervention group was superior to that of the endoscopic diagnostic group, and the advantage was more prominent when compared with the conventional group, whose healing velocity was only one-third of its counterpart.

#### Landmark Analysis for the First 30 Days

Patients in the systematic endoscopic group had significantly lower rates of death than those in the endoscopic diagnostic group and the conventional group, while no obvious difference in fatality was observed between the endoscopic diagnostic group and the conventional group. In the weighted Cox proportional hazard regression model, the adjusted hazard ratio (HR) for healing in the endoscopic intervention group compared with the conventional group was 1.94 (95% CI, 0.68-5.51; P=.038), and that in the diagnostic group compared with the conventional group was 1.40 (95% CI, 0.66-3.00; P=.021). In this analysis, the differences among the 3 groups were not statistically significant with regard to healing (**Figures 3** and **4**).

#### Landmark Analysis for 30-60 Days

Again, the possibility of death in the systematic interventional group was significantly lower than that of the conventional group; meanwhile, a similar advantage was found in the endoscopic diagnostic group when compared with the conventional group. Moreover, when compared with the traditional group, the endoscopic intervention group and endoscopic diagnostic group showed not only a significant reduction in the mortality rate but also a statistically significant increase in the recovery rate; the hazard ratios for healing were 3.86 (95% CI, 1.93-7.75; P<0.001) and 2.57 (95% CI, 1.56-4.25; P<0.001), respectively (**Figures 3** and **4**).

#### Landmark Analysis for 60-90 Days

A lower mortality rate was found in the endoscopic diagnostic group than in the conventional group, which had 4 fatal cases, yet the mortality rate seemed to be higher in endoscopic interventional group than in the remaining two groups; notably, only 5 patients were in the endoscopic interventional group during this period, which should be taken into consideration. During this period, the HRs for healing were 2.69 (95% CI, 0.89-8.10; P=0.08) in the endoscopic interventional group and 1.50 (95% CI, 0.71-3.18; P=0.29) in the endoscopic diagnostic group when compared with their counterparts (**Figures 3** and **4**).

#### Landmark Analysis for Post-90 Days

It should be noted that all patients in the systematic endoscopic group reached the study endpoints. As illustrated in **Figures 3** and **4**, during the period 3 months after surgery, the endoscopic diagnostic group and conventional group healed at very similar speeds, and the mortality rates were 3/11 (27.3%) and 6/28 (21.4%), respectively.

##### Mortality and Complications:

Of the 203 enrolled patients, there were 20 (21.28%) fatal cases among the 94 patients in the conventional group, 9 (10.34%) fatal cases among the 87 patients in the endoscopic diagnostic group and 1 (4.55%) fatal case among the 22 cases in the endoscopic intervention group; this difference was statistically significant (Fisher exact test, *P*=0.049<0.05).

Regarding compilations, 24 (25.53%) complications occurred in the 94 patients in the conventional group, 19 (21.84%) occurred in the 87 patients in the endoscopic diagnostic group, and 1 (4.55%) occurred in the 22 patients in the endoscopic intervention group, but the differences among the three groups were not statistically significant (Fisher’s exact test, *P*=0.089>0.05).

Therefore, in conclusion, 30 patients died, and 44 patients developed EAL-related complications. The overall mortality and complication rates were 14.78% and 21.67%, respectively. The overall survival rate was 78.7% (95% CI: 70.3 to 87.2%) in the conventional management group, 89.7% (95% CI: 83.1 to 96.2%) in the endoscopic diagnostic group and 95.5% (95% CI: 86.0 to 100%) in the systematic endoscopic intervention group (**Table 5**).

**Table 5:** Mortality and Complications of the 203 patients with EAL.

Groups	No. of patients	Mortality	Complications			Total
	(N=203)		AS	SF	H	
Conventional management	94	20(21.28%)	19(20.21%)	2(2.13%)	3(3.19%)	24(25.53%)
Endoscopic diagnosis	87	9(10.34%)	14(16.09%)	3(3.45%)	2(2.30%)	19(21.84%)
Systematic endoscopic intervention	22	1(4.55%)	0	0	1(4.55%)	1(4.55%)
Total	203	30(14.78%)	33(16.26%)	5(2.46%)	6(2.96%)	44(21.67%)

AS, anastomotic stenosis; SF, sinus formation; H, hemorrhage.

## Discussion

Post-esophagectomy anastomotic leakage or fistula is a serious and common complication in patients with esophageal carcinoma (2–5, 7, 9–11). Over the past decade, few studies have adequately assessed and evaluated the status of endoscopic technology for the diagnosis and treatment of EAL, and to the best of our knowledge, this paper is the first to discuss the relationship between EAL healing and the timeframe in which healing occurred, not just whether it was healed or not. We found that patients with EAL after endoscopic intervention may have the fastest healing speed at 30-60 days (1-2 months) after surgery based on the landmark analysis results (compared with the conventional management group and the endoscopic diagnosis group, HR values were 3.86 and 2.57, respectively). This may provide a reference to help clinicians make better clinical decisions at different time periods.

EAL can affect the operative efficacy of esophageal cancer, prolong hospital stays and increase postoperative mortality (2, 5–11). EAL can even impair patient quality of life, long-term survival of esophageal cancer and subsequent treatment of esophageal masses using strategies such as adjuvant chemoradiotherapy (2, 6, 8, 10, 25). Finally, because EAL potentially causes subsequent critical postoperative complications, such as intrathoracic abscess, tracheoesophageal fistula and hemorrhage, both predicting and treating EAL are clinically significant issues. Therefore, it is of vital importance to explore a safe and effective treatment model for EAL. The present study focused on the role of systematic endoscopic intervention in postoperative EAL detection and rehabilitation. Our study included patients with EAL following surgery for esophageal cancer at a specialized cancer center, representing a larger, more homogenous patient population.

Given the high incidence of anastomotic leakage and the severe harm it causes, most centers prefer to assess the anastomosis diagnostically before starting oral intake after esophagectomy. The use of endoscopy, however, has been questioned due to the theoretical threat of disrupting the anastomosis or aggravating EAL (17–19). At present, many surgeons in China still pay little attention to or are reluctant to attempt to address EAL by endoscopic means for fear of the possible complications mentioned above. Our findings show that properly performed endoscopic intervention does not cause injury to the anastomosis, and a certain number of studies have proven the safety of endoscopy (14–16); although an intraluminal pressure greater than 80 cmH2O is known to be required to disrupt the anastomosis, the intraluminal maximum insufflation at the anastomosis never exceeds 9 cmH2O and thus rarely disturbs blood flow in the conduit (14, 26–28).

Patients who underwent endoscopic diagnosis and/or intervention had lower probabilities of death and complications than the conventional group in our study (**Table 5**). It was found that the overall mortality was 14.78%. By comparison, the mortality rates presented in previous studies have ranged from 2.1% to 35.7% (2, 8, 9, 11, 29, 30).

Patients in the endoscopic diagnostic group vs. conventional group had a lower risk of death (odds ratio (OR) =0.43; 95% CI, 0.18-1.00); after adjustment by the Bonferroni method, however, there were no statistically significant differences between the groups with regard to mortality (*P*=0.067>0.01667). Patients in the endoscopic intervention group vs. conventional group also had a lower risk of death (odds ratio (OR) =0.18; 95% CI, 0.02-1.40), but again, no statistically significant difference was observed (*P*=0.119>0.01667). Regarding compilations, 24 (25.53%) complications occurred in the 94 patients in the conventional group, 19 (21.84%) occurred in the 87 patients in the endoscopic diagnostic group, and 1 (4.55%) occurred in the 22 patients in the endoscopic intervention group, but the differences among the three groups were not statistically significant (Fisher’s exact test, *P*=0.089>0.05).

Moreover, the sensitivity and specificity of endoscopic assessment were superior to those of traditional methods. In our study, the sensitivity of traditional diagnostic methods was 75.81%, close to the previously reported CT diagnostic sensitivity of 71.4-80% (5, 12, 13, 26, 27), which is unsatisfactory. While endoscopy not only correctly identified the 5 false-positive patients evaluated by radiological contrast study but also determined 30 leaks that were missed in the radiological contrast study, and both the reported sensitivity and specificity of endoscopic diagnosis could reach 100% (16).

Additionally, the procedure is convenient, as it can be conducted at the bedside, even for patients on ventilation, without worsening an existing EAL. More remarkably, endoscopy is the only approach with the capacity to determine the viability of the gastric conduit and to grade the EAL according to the results of endoscopic observation, which will be highly valuable in making more accurate clinical decisions based on each individual, including the adjustment of the drainage strategy, the need for surgical treatment, the use of antibiotic regimens, adequate nutritional support, and so on. In summary, endoscopic diagnosis offers the advantages of possibly avoiding repetitive examinations, aiding in early diagnosis, guiding further treatments, improving the sensitivity and specificity, and reducing complications, which could make the treatment process more smoothly and accurately, and then enable patients to achieve better clinical outcomes.

**Figure 5:**
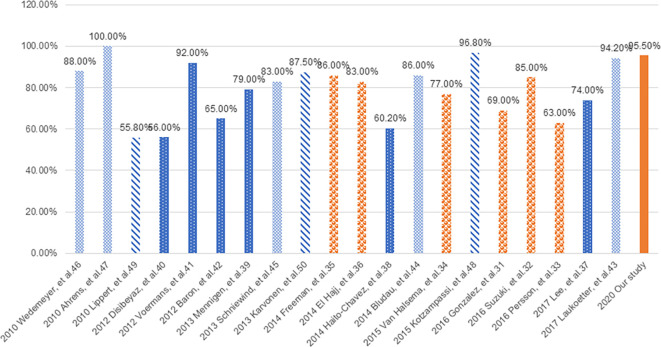
Healing rates of EAL about part of previous studies.

With regard to the treatment, although we were interested in determining whether the EAL heals, we were more curious about when. To our knowledge, this paper is the first to discuss the outcome of anastomotic leaks in association with healing time rather than whether it healed based on the results of landmark analysis. Previous studies have reported 55.8-100.0% healing rates for EAL when treated with endoscopic strategies (31–50), while our research suggests that the healing rates could reach up to 95.5% if endoscopic management methods were implemented, and the number would still be near 90% if only endoscopic diagnosis was implemented [**Table 2** and **Figure 5** (31–50)].

Moreover, we elucidated the actual healing time and successfully identified two statistically significant independent factors associated with the time needed for healing EAL, of which different endoscopic strategies were included (**Table 1**).

Regarding how to reduce the healing time, endoscopy offered a satisfactory result. The goal of the landmark analysis method was to estimate the healing probabilities in each group at the landmark time in an unbiased way (22–24).The landmark analysis revealed that once the endoscopic intervention was administered, the superiority of endoscopic intervention compared with conventional management persisted until the leisure healed, and this advantage is most pronounced 1 to 2 months after surgery, which indicated that early intervention is of vital importance to the recovery process of EAL. Patients with EAL were found to heal faster than conservative patients even when only endoscopic diagnosis was conducted without systematic endoscopic intervention at the early stage; however, the superiority of the endoscopic diagnostic group compared with the traditional group before 90 days of follow-up was lost after 90 days. Of course, the healing time of EAL would be shorter if endoscopic intervention was added. Interestingly, it was found that if the patients in the endoscopic diagnosis group did not achieve clinical cure at an early stage when there was a healing advantage, their merits of rapid rehabilitation would nowhere to be seem as time goes by, put it another way, they would be found to have similar clinical outcomes as those in the conventional group at later stages of the follow-up, which provides a new perspective on the importance of early diagnosis of EAL, and suggests that patients with EAL may benefit from remedial endoscopic managements.

To summarize, our study provides new evidence that endoscopic therapy can offer an important prognostic benefit to EAL patients. Endoscopic intervention could be considered superior to other regimens in managing anastomotic leakage at any period after esophagectomy. The landmark analysis results suggested that for EAL rehabilitation, endoscopic therapy can be attempted as a remedial measure at any period, even if endoscopic intervention was not employed at the early stage, since remedial endoscopy could shorten the healing time of EAL.

In terms of the clinical application of the results of this study, it is important to take into account the merits of a shorter healing time. Shortening the time needed for EAL healing has some potential clinical advantages, including reducing the incidence of subsequent critical postoperative complications and decreasing the cost of hospitalization due to the shortened hospitalization period. In addition, a shorter healing time allows for smoother coordination of the administration of adjuvant therapy when patients have cancers for which adjuvant therapy is indispensable.

The present study has several limitations. First, endoscopic vacuum-assisted closure (E-VAC) therapy was not carried out in our hospital; more specifically, E-VAC technology has not been widely used throughout China. E-VAC technology was first introduced in 2008 by Weidenhagen et al. (51) and has been proven to be safe and effective in some studies, with encouraging healing rates (93.3-93.5%) (52, 53). We look forward to using E-VAC technology in our hospital to help patients who have suffered from EAL. The second limitation is that the data for the present study were from a single institution, which may produce some bias in the preoperative management of patients, such as operative methods. In the future, these results should be validated in a multi−institutional, prospective, randomized, controlled trial using certain criteria, as mentioned above.

In summary, the results of this study suggest that systematic endoscopic intervention is an effective and safe method for the diagnosis and treatment of postsurgical leaks. This intervention leads to higher success rates and faster anastomotic healing and has the potential to reduce overall mortality. These findings could provide guidance for clinicians to promote earlier recovery from EAL.

## Data Availability Statement

The raw data supporting the conclusions of this article will be made available by the authors, without undue reservation.

## Ethics Statement

Ethical review and approval were not required for the study on human participants in accordance with the local legislation and institutional requirements. Written informed consent for participation was not required for this study in accordance with the national legislation and the institutional requirements.

## Author Contributions

These authors contributed equally and share the first authorship. All authors contributed to the article and approved the submitted version.

## Funding

This study was supported by the grant from National Key R&D Program of China (2018YFC0910600). The funding body had no role in the design of the study and collection, analysis, and interpretation of data and in writing the manuscript.

## Conflict of Interest

The authors declare that the research was conducted in the absence of any commercial or financial relationships that could be construed as a potential conflict of interest.
